# In Vitro Antitrypanosomal and Antibacterial Activity of Aqueous, Hydroethanolic and Ethanolic Extracts of *Rumex obtusifolius* L. Leaf and Root

**DOI:** 10.1111/bcpt.70230

**Published:** 2026-04-08

**Authors:** Gabriela K. Borges, Gabriella B. Das Neves, Letícia Reichardt, Ubirajara Maciel da Costa, Luiz C. Miletti, Amanda L. Bastos‐Pereira

**Affiliations:** ^1^ Department of Veterinary Medicine, Center for Agricultural Sciences (CAV) State University of Santa Catarina (UDESC) Lages Santa Catarina Brazil; ^2^ Department of Animal and Food Production, Center for Agricultural Sciences (CAV) State University of Santa Catarina (UDESC) Lages Santa Catarina Brazil

**Keywords:** antitrypanosomal, flow cytometry, *Mus musculus*, *Rumex obtusifolius*, *Trypanosoma evansi*

## Abstract

Plant extracts are an important raw material for the development of new drugs. *Rumex obtusifolius* L., popularly known as ‘bitter dock’, has significant pharmacological properties. This study evaluated the in vitro antitrypanosomal activity of aqueous, hydroethanolic and ethanolic extracts from the leaf and roots of *R. obtusifolius* L. against *Trypanosoma evansi*, as well as their antibacterial activity against *Escherichia coli* and *Streptococcus aureus*. Antitrypanosomal assays were performed using extract concentrations of 0.5%, 1% and 2% and analysed by flow cytometry (necrosis). The antimicrobial activity was assessed at 1–100 mg/mL. Cytotoxicity was evaluated in mammalian cells for extracts active against *T. evansi* . The hydroethanolic and ethanolic leaf extracts exhibited significant anti‐ *T. evansi* activity, inducing necrosis rates of 64.6% and 63.7%, respectively. Besides, treatment with the ethanolic leaf extract (2%) of *R. obtusifolius* reduced cell viability to 14.3% after 24 h, whereas the hydroethanolic extract (2%) exhibited lower cytotoxicity, with cell viability remaining at approximately 65%–70%. None of the extracts exhibited antibacterial activity. In addition, our findings demonstrate a previously unreported pharmacological property of *R. obtusifolius* : the antitrypanosomal activity of its hydroethanolic and ethanolic extracts against *T. evansi* . Nevertheless, further studies are required to elucidate the mechanisms of action and to confirm a possible in vivo antitrypanosomal efficacy of these extracts.

## Plain Language Summary

This study investigated the pharmacological properties of *Rumex obtusifolius* L. (bitter dock) extracts, focusing on their in vitro antitrypanosomal activity against *T. evansi* and antibacterial effects against *E. coli* and *S. aureus* . Aqueous, hydroethanolic and ethanolic extracts from leaves and roots were tested at various concentrations. Results showed that hydroethanolic and ethanolic extracts from leaves exhibited anti‐ *T. evansi* activity, causing necrosis in parasites. None of the extracts effectively inhibited bacterial growth. Additionally, the cytotoxic potential of the extracts was assessed in mammalian cells, highlighting the need for further research on their therapeutic applications.

## Graphical abstract

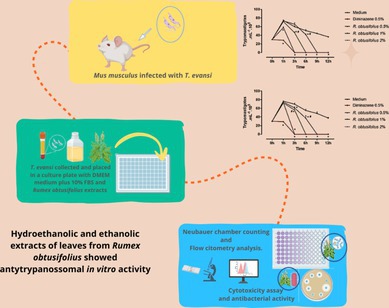

## 1 Introduction

‘Surra’ is a trypanosomiasis caused by the flagellate protozoan *Trypanosoma evansi*, which affects wild and domestic animals. Its treatment is mainly based on the administration of diminazene aceturate, a chemotherapeutic that may cause hepatotoxicity and lead to parasite resistance [1]. New cases of *T. evansi* have been reported for the first time in different locations [2, 3, 4], and human infections caused by this agent have already been reported [5, 6, 7]. Ineffective treatment, lack of specific diagnosis and vector control make the need for new therapeutic alternatives urgent. Another globally comprehensive problem is bacterial resistance, which is attributed, in part, to the overuse of antibiotics [8].

Beyond that, the pharmaceutical industry has not been promoting the discovery and development of new drugs or active compounds, and it is extremely important to innovate in this area [9, 10]. Antiparasitic and antibacterial activities constitute pressing therapeutic challenges. Both issues, antiparasitic and antibacterial actions, represent urgent therapeutic challenges. In this context, plant‐derived compounds might emerge as a vital frontier for the development of novel therapeutic agents.

Plant species are a rich source of bioactive compounds of pharmaceutical interest. The Rumex genus may exhibit multiple pharmacological activities, including anti‐inflammatory, antioxidant, antibacteria, antitumor, antiviral and antiparasitic [11, 12, 13, 14]. *Rumex obtusifolius* L. (‘bitter dock’) is an edible and medicinal plant widespread in temperate regions, including southern Brazil. It is traditionally used for its diverse pharmacological properties, such as antibacterial, antioxidant, antiviral and antifungal activities [15, 16]. *R. obtusifolius* presents a noteworthy phytochemical profile, comprising anthracene derivatives, flavonoids and procyanidins, predominantly identified in its aerial part and roots [17, 18].

Based on these considerations, this study aimed to evaluate the antitrypanosomal and antibacterial properties of *R. obtusifolius* extracts against *T. evansi*, *Escherichia coli and Streptococcus aureus*.

## 2 Material and Methods

### 2.1 Botanical Material and Extract Preparation

Samples of *R. obtusifolius* were identified and collected in the experimental organic area (approximated location 27°48′18.4″ S 50°20′18.4″ W), from Federal Institute of Education, Science and Technology of Santa Catarina State (Lages City, Santa Catarina State, Brazil), in the second semester of 2017 (spring season). The plant was registered at the University Herbarium (LUSC, UDESC; voucher #10196), corresponding to the taxonomic classification of *R. obtusifolius* . Leaves were separated from the root and washed with water, homogenized in a mechanical homogenizer and stored in an industrial freezer (−18°C ± 1°C). Extracts were made with fresh samples and experiments were performed using aqueous, hydroethanolic or ethanolic solutions (1:10 w/v), according to Sganzerla et al., 2019 [19]. The study was conducted in accordance with the ‘Basic & Clinical Pharmacology & Toxicology policy for studies involving natural products, traditional Chinese medicine and systems pharmacology’ along with a reference to the BCPT policy [20].

The moisture (105°C, 24 h) (method 934.06) and ashes (550°C, 12 h) (method 923.03) content was determined according to the Association of Official Analytical Chemistry [21]. All extracts were filtered using a syringe and nitrocellulose filter (20 μm) and stored in an ultrafreezer (−180°C) until use.

### 2.2 In Vitro Analysis of Bioactive Compounds

Total phenolic content (TPC) was determined according to Sganzerla et al. [19]. Total flavonoid content (TFC) and total flavonols (TF) were determined according to the methods of Zhishen et al. [22] with modifications. All measurements were carried out in triplicate (*n* = 3). Absorbance readings were obtained at 765 nm (TPC), 510 nm (TFC) and 415 nm (TF). Calibration curves were prepared using gallic acid (GAE) and quercetin as standards (*R* ^2^ > 0.99). Results were expressed on a fresh weight basis.

### 2.3 In Vitro Antioxidant Activity Determination

To analyse antioxidant activity, three methods were performed: removal of the free radical DPPH (2,2‐Diphenyl‐1‐picrylhydrazyl), removal of the free radical ABTS (acid 2,2′‐azino‐bis‐3‐ethylbenzotiazoline‐6‐sulfonic); and the ferric reducing antioxidant power (FRAP) assay. Trolox was used as a standard, and the results are expressed in mg of Trolox equivalent antioxidant capacity (TEAC) per 100 g of root or leaf in fresh matter (mg TEAC 100 g^−1^). All antioxidant activity analyses and bioactive compounds were described by Sganzerla et al. [19].

### 2.4 Animals and Ethics Statement

27 animals were used to develop *T. evansi* infection and parasites in an axenic culture, as described below. Male Swiss mice ( *M. musculus* ) (40–55 g; 60 days old), from a regulated breeding facility (Federal University of Santa Catarina, Florianopolis, Brazil) were maintained under standard conditions (animal room with an atmospheric micro‐insulation system, temperature 22° ± 2°C, 12 h light/dark cycle), with free access to food and water, housed up to 10 mice each. Polyvinylchloride pipes and paper nests were used as environmental enrichment. The heterologous experimental infection is largely investigated [23] in many species, and the mouse was chosen because of its size and easier management. The study was conducted in accordance with the Basic & Clinical Pharmacology & Toxicology policy for experimental and clinical studies [24] and clear humane endpoints were established to safeguard animal welfare. The authors followed and filled the ARRIVE guidelines, necessary for this study's publication. The use of mice in the experiments was approved by the local Ethics Committee on the Use of Animals (CEUA UDESC; under #23660606919), and strictly guided by the rules of the Brazilian Directive for the care and use of animals in teaching or scientific research activities (RN 30/2016—CONCEA).

### 2.5 In Vitro Evaluation of Extracts Against *T. evansi*

Trypomastigotes were obtained from 10% glycerol‐preserved cultures that were stored at −80°C [25]. For each experiment, one animal was inoculated with 0.1 mL of cryopreserved culture (10^6^ parasites.mL^−1^), diluted with phosphate‐buffered saline (PBS) enriched with 60% glucose. When the animal showed high parasitemia (50 parasites per field under a 40‐fold objective optical microscope), it was anaesthetized (ketamine 90 mg.kg^−1^ plus xylazine 7.5 mg/kg, IP) and its blood was collected via cardiac puncture. The euthanasia was completed by cervical dislocation.

Purified parasites (3 × 10^5^ parasites/mL) that were resuspended in PBS‐glucose buffer, were added to the culture and exposed to various concentrations of *R. obtusifolius*. Culture medium was prepared using minimal essential medium (MEM, M4655 Sigma‐Aldrich). Each extract (0.5%, 1% or 2%, equivalent to 5, 10 or 20 mg/mL), medium (negative control) or diminazen 0.5% (positive control) was added at 25 μL per well, plus 270 μL of medium with parasites (3 × 10^5^ parasites/mL) per well. Samples were kept in an incubator at 37°C with 5% CO_2_. Treatments were carried out in triplicate (three wells per treatment and three independent experiments), and the number of parasites was recorded at 1, 3, 6, 9 and 12 h after the beginning of the experiment, using a Neubauer chamber (Blaubrand). Parasite viability was assessed based on the presence or absence of motility.

### 2.6 Cell Viability Assessment and Necrosis Biomarking

Purified parasites (3 × 10^5^ parasites/mL), similarly to above, were added to the culture and exposed to *R. obtusifolius* . For this experiment, the extracts chosen were the best results obtained in the Neubauer counting experiment (hydroethanolic and ethanolic leaf extracts at 2%). Furthermore, to analyse the negative controls (hydroethanolic and ethanolic) in flow cytometry, 270 μL of medium (MEM, M4655 Sigma‐Aldrich) was used per well, plus 25 μL of 100% ethanol for ethanolic control and 25 μL (of which, 7.55 μL of 100% ethanol and 17.5 μL of deionized water) to hydroethanolic control.

All samples were kept in an incubator at 37°C with 5% CO_2_ for 4 h and tested (triplicates) on a flow cytometer (BD Accuri C6).

To identify the cell location of *T. evansi* , SSC/FSC and FL2 (standard optical filter 585/40 nm) parameters were used to determine the fluorescence intensity of live and dead cells in the fluorescence channel. To analyse the necrosis of parasites subjected to the action of *R. obtusifolius* , 0.1% propidium iodide (P4170, Sigma‐Aldrich) was used since it only penetrates necrotic cells, especially via membrane disruption [25].

Flow cytometry analyses were performed with 80 000 events acquired at a slow flow rate. Data were analysed using BD Accuri C6 Software and graphs generated using FlowJo software (v.10.9.0).

### 2.7 In Vitro Cytotoxicity Assay

Extracts from leaf and root of *R. obtusifolius* , at 2% (20 mg/mL) were added to VERO cell line (10^4^ cells/mL)^,^ cultivated in DMEM (D5523, Sigma‐Aldrich) supplemented with 100 μL/well of 10% FBS (F7524, Sigma‐Aldrich), at 37°C with 5% CO_2_. Two control groups were used: (i) blank control (medium without cells) and (ii) negative control (medium with untreated cells). After 24 h, an MTT (3‐[4, 5‐dimethylthiazol‐2‐yl]‐2, 5 diphenyl tetrazolium bromide) assay was performed: the contents of each well were removed, replaced by 100 μL of fresh medium, 10 μL of a 12 mM MTT (M2003, Sigma‐Aldrich) solution and incubated (37°C) for more 4 h. Thereafter, 85 μL of the volume of each well was removed and 50 μL of dimethyl sulphoxide, DMSO (472 301, Sigma‐Aldrich) was added and incubated (37°C) for 10 min. Each well was homogenized, and the plate was placed on a shaker for 5 min to solubilize formazan crystals, which are the marker of cell viability and colouring. Absorbance was measured at 490 nm (Mindray, MR‐96A, Hamburg, Germany), and cellular viability was calculated using the following equation (in percentage): ((ODa—ODb) / (ODcp—ODb)) × 100, where ODa is the optical density of formazan production of the sample; ODb is the optical density of the blank (DMEM without cells); and ODcp is the optical density of the positive control (cells cultured with DMEM and 10% FBS). This experiment was conducted three times, and each treatment was performed in triplicates.

### 2.8 In Vitro Antimicrobial Activity

To assess antimicrobial activity, a disk diffusion test was performed using standard microorganisms ( *Staphylococcus aureus* or *E. coli* ). Microorganisms were replicated in BHI medium (K25–1048, Kasvi) and incubated at 37°C for 24 h before the experiment. To prepare the inoculum, cultivated bacteria were transferred to test tubes containing 2 mL of sterile saline until a turbidity equivalent to half of the 1.0 MacFarland standard was obtained. The inoculum was applied to Mueller Hinton agar plates (K25–1058, Kasvi) with a swab, and the discs soaked with extracts (aqueous, hydroethanolic or ethanolic extracts of the leaf or roots of *R. obtusifolius* ) were inserted in 6 mm filter paper discs. Then, 20 μL of each extract was added at concentrations of 1, 3, 10, 30 or 100 mg/mL. Vehicles containing distilled water, hydroethanolic and ethanolic solutions were used as negative controls. The positive controls were 25 μg of sulfazotrim and 30 μg of tetracycline (both from Laborclin). Plates were incubated at 37°C for 24 h in order for the diameters of inhibition to be observed. The assays were performed in triplicate.

### 2.9 Statistical Analysis

Data were statistically analysed using GraphPad Prism Software 7.0, previously submitted to Shapiro–Wilk normality test, and then to one‐way or two‐way ANOVA statistical tests and post hoc Tukey or Bonferroni tests, as all data were considered parametric. Statistical significance was set at *p* < 0.05. For the counting of parasites results, IC_50_ was assessed through nonlinear regression.

For flow cytometry, data were analysed using BD Accuri C6 Software and graphs generated using FlowJo software (v.10.9.0). Two parameters were analysed: parasite counting, obtained from the FSC/SSC histogram and generated from the light scattering properties of the parasite and the percentage of events with fluorescence intensity in the FL2 channel of necrotic cells. Extracts were considered to possess anti‐ *T. evansi* activity when the qualitative analysis induced cell necrosis and the statistical analysis was significant.

## 3 Results

### 3.1 In Vitro Analysis of Bioactive Compounds

The results of the chemical analysis of the TPC and total flavonoids, as well as those of the antioxidant activity of the aqueous, hydroethanolic and ethanolic extracts of leaf and roots of *R. obtusifolius* , are shown in Table 1.

**TABLE 1:** Total phenolic content (TPC), total flavonoids (TF) and in vitro antioxidant activity (DPPH, ABTS and FRAP assays) of aqueous, hydroethanolic and ethanolic extracts of leaf and root of *Rumex obtusifolius.* Results are expressed as mean ± standard deviation. Different letters in each column after standard deviation represent a significant difference by Tukey's test (*p* < 0.05). TPC: Total Phenolic Content; TF: total flavonols. 1 mg of Gallic Acid Equivalent (GAE) 100 g^−1^; 2 mg of quercetin equivalent (QE) 100 g^−1^.

*R. obtusifolius*	Extract	TPC	TF	FRAP	DPPH	ABTS
Leaves	Aqueous	19.62 ± 0.18^e^	2.72 ± 0.28^e^	46.22 ± 2.06^e^	32.26 ± 0.84^c^	17.05 ± 1.27^e^
	Hydroethanolic	234.66 ± 9.06^d^	263.52 ± 2.75^d^	1472.19 ± 192.38^d^	1383.33 ± 50.50^b^	633.24 ± 6.37^d^
	Ethanolic	387.25 ± 3.63^c^	403.78 ± 2.75^c^	2953.46 ± 106.88^c^	1859.52 ± 16.83^b^	938.72 ± 6.35^c^
Roots	Aqueous	17.05 ± 0.17^e^	8.94 ± 0.45^e^	195.59 ± 27.79^e^	84.05 ± 5.05^c^	67.82 ± 0.63^e^
	Hydroethanolic	980.95 ± 1.81ª	155.08 ± 1.83ª	13579.24 ± 342.01^a^	3800 ± 303.05^a^	2695.29 ± 888.94
	Ethanolic	573.1 ± 23.57^b^	108.97 ± 0.92^b^	5704.37 ± 106.88^b^	3311.90 ± 117.85	1859.65 ± 12.70

The extract which showed the highest amount of TPC per mg of gallic acid was from the hydroethanolic extract from the root of *R. obtusifolius* , with 980.95 mg GAE/100 g. The hydroethanolic root extract has also shown the highest values for ABTS (2695.29 ± 88.94 mg TEAC/100 g fresh weight) and FRAP (13579.24 ± 342.01 mg TEAC/100 g fresh weight), being significantly higher than all other extracts (*p* < 0.05). The lowest amount of TPC was from the aqueous extract from the root of *R. obtusifolius* (17.05 mg GAE/100 g of extract).

Leaf extracts showed lower antioxidant activity than root extracts. Among leaf samples, the ethanolic extract displayed the highest FRAP value (2953.46 ± 106.88 mg TEAC/100 g fresh weight), followed by the hydroethanolic extract (1472.19 ± 192.38 mg TEAC/100 g fresh weight). The ethanolic leaf extract also exhibited significantly higher TPC (387.25 ± 3.63 mg GAE/100 g fresh weight) and TFC (403.78 ± 2.75 mg QE/100 g fresh weight) compared with the hydroethanolic extract (*p* < 0.05).

### 3.2 In Vitro Evaluation of Extracts Against *T. evansi*

**FIGURE 1:**
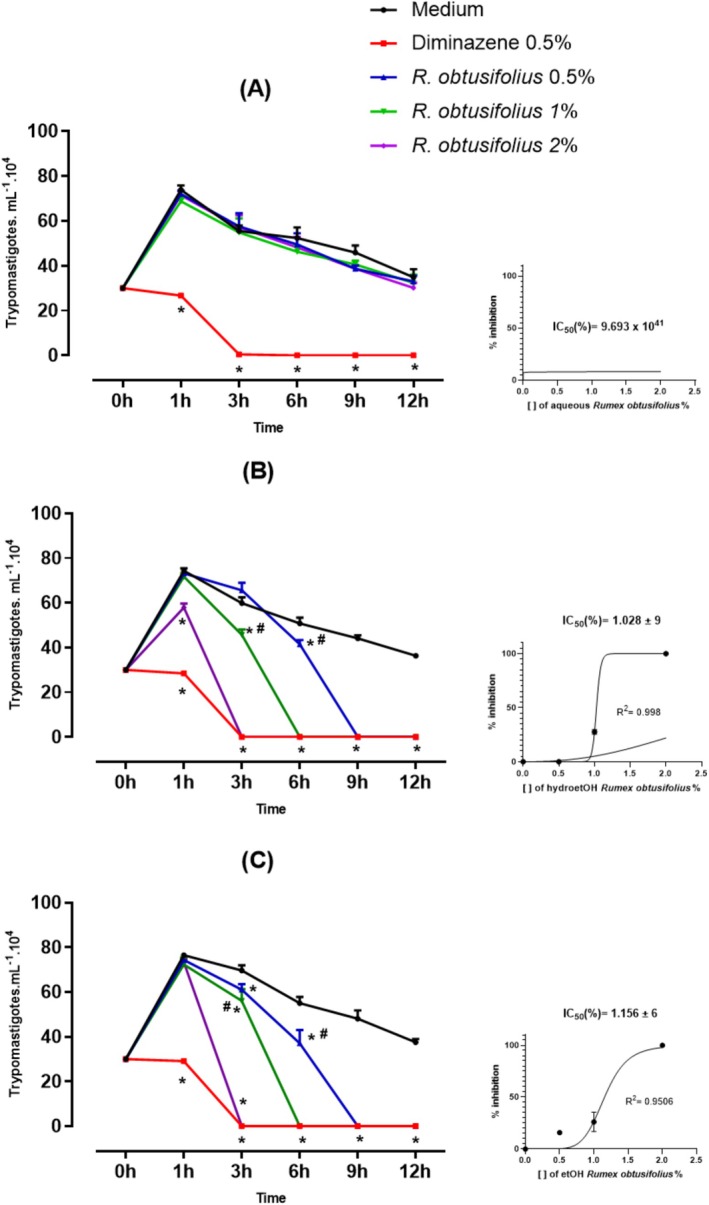
Antitrypanosomal activity in vitro against *Trypanosoma evansi* at concentrations of 0.5, 1 and 2% of aqueous (A), hydroethanolic (B) and ethanolic (C) extract of leaves from *Rumex obtusifolius* , compared to negative control (culture medium) and positive control (0.5% diminazene). Details show the IC_50_values for each extract, in percentage ± degrees of freedom. Symbols * show values of *p* < 0.05 compared to the negative control and the symbols # show values of *p* < 0.05 compared to the positive control. Results are expressed as mean ± standard deviation. Two‐way ANOVA, followed by Tukey. For IC_50_ calculation, nonlinear regression.

**FIGURE 2:**
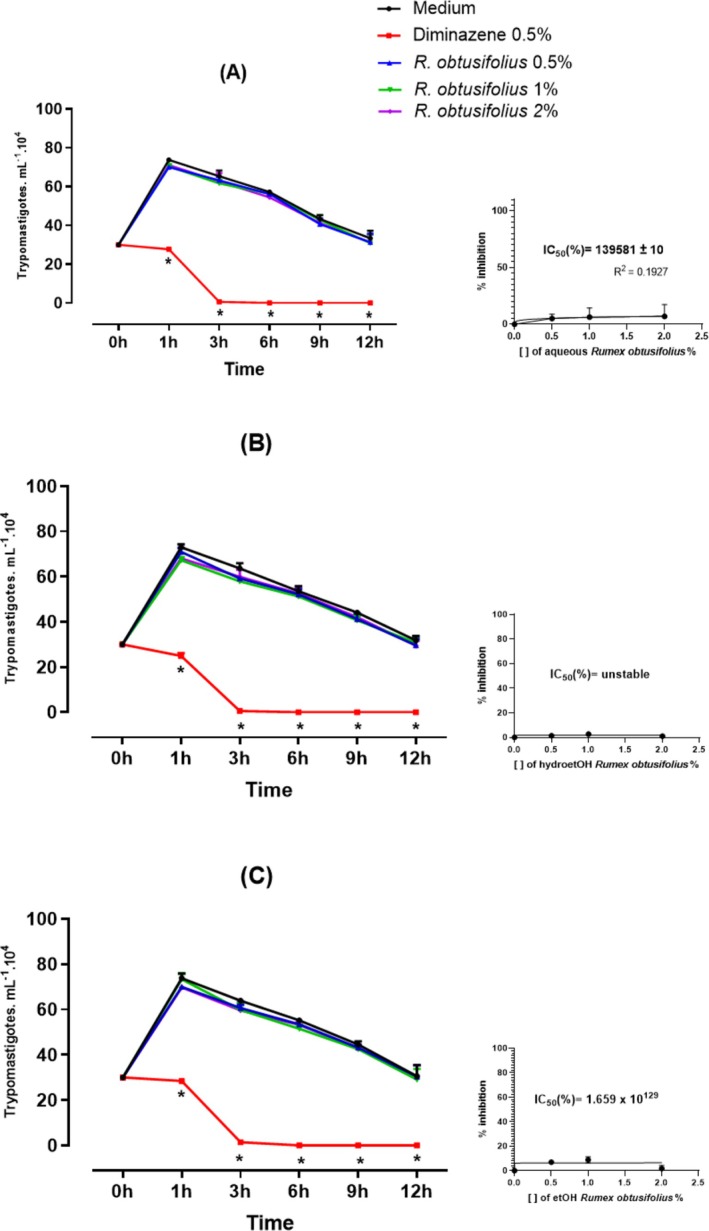
Antitrypanosomal activity in vitro against *Trypanosoma evansi* at concentrations of 0.5%, 1% and 2% of aqueous (A), hydroethanolic (B) and ethanolic (C) extract of roots of *Rumex obtusifolius* , negative control (culture medium) and positive control (0.5% diminazene). Details show the IC_50_values for each extract, in percentage ± degrees of freedom. Symbols * show values of *p* < 0.05 compared to the negative control. Results are expressed as mean± standard deviation. Two‐way ANOVA, followed by Tukey. For IC_50_ calculation, nonlinear regression.

The antitrypanosomal activity of the extracts is shown in Figures 1 and 2. During the parasite culture experiment, the leaves aqueous extract *R. obtusifolius* (Figure 1A), at all concentrations, could not demonstrate anti‐ *T. evansi* activity. On the other hand, the hydroethanolic extract (Figure 1B), at its highest concentration (2%), was able to slightly reduce the number of parasites in the first hour (57.96 ± 2.895) of the experiment, compared to the negative control (74.29 ± 1.942). The same extract was also able to eliminate all the parasites after 3 h of culture, compared to the medium (59.88 ± 4.444). After 6 h, the extract at 1% eliminated all parasites (control group 50.79 ± 4.563), and 0.5% of this extract eliminated all parasites after 9 h (control group 44.13 ± 2.382).

Ethanolic extract from leaves of *R. obtusifolius* (Figure 1C) showed inhibition response at 0.5%, 1% and 2%, with parasites eliminated after 9, 6 and 3 h of the experiment, respectively, compared to the negative control (48.08 ± 6467 at 9; 55.00 ± 4.918 at 6 and 69.71 ± 3.908 at 3 h). The highest concentration (2%) was able to completely eliminate parasites after 3 h of the experiment. An IC_50_ was calculated for the third hour of analysis, being 1.028% ± 9 and 1.156% ± 6 for the hydroethanolic and ethanolic extracts, respectively.

In contrast to that observed in all leaves extracts of *R. obtusifolius* , the extracts from the roots (Figure 2A,B,C) did not show antitrypanosomal activity against *T. evansi* . The IC_50_ values were too high or not possible to be calculated (unstable).

Considering the aforementioned results, hydroethanolic and ethanolic extracts from the leaf of *R. obtusifolius* continued to be evaluated in the subsequent experiments, since they have shown anti‐ *T. evansi* activity in vitro.

### 3.3 Cell Viability Assessment and Necrosis Biomarking

**FIGURE 3:**
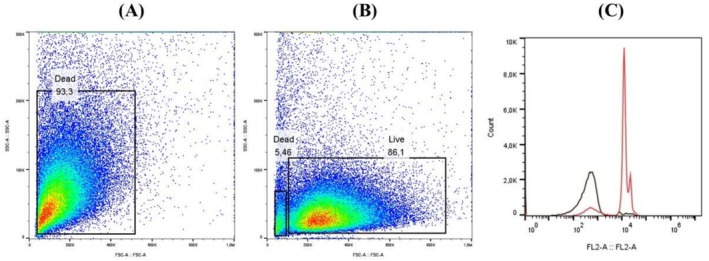
(A) SSC‐A/FSC‐A: Purified dead parasites with ethanol; it was possible to. (B) SSC‐A/FSC‐A: Purified live parasites. The graphic shows the population of *T. evansi* delimited according to cell size and complexity. (C) Count/FL2:A (propidium iodide): Overlapping plot between fluorescence graphs of living (black line) and dead (red line).

Figure 3A shows the percentage of dead *T. evansi* (93.3%) with 100% ethanol, identifying cell membrane disruption, where it is possible to observe the loss of membrane integrity. Figure 3B shows purified live parasites (86.1%). Both figures demonstrated the effectiveness of flow cytometry technique. The increase in fluorescence in ethanol‐treated (Figure 3C, red line) was used as criterion to determine cell necrosis compared with control sample (untreated, black line).

**FIGURE 4:**
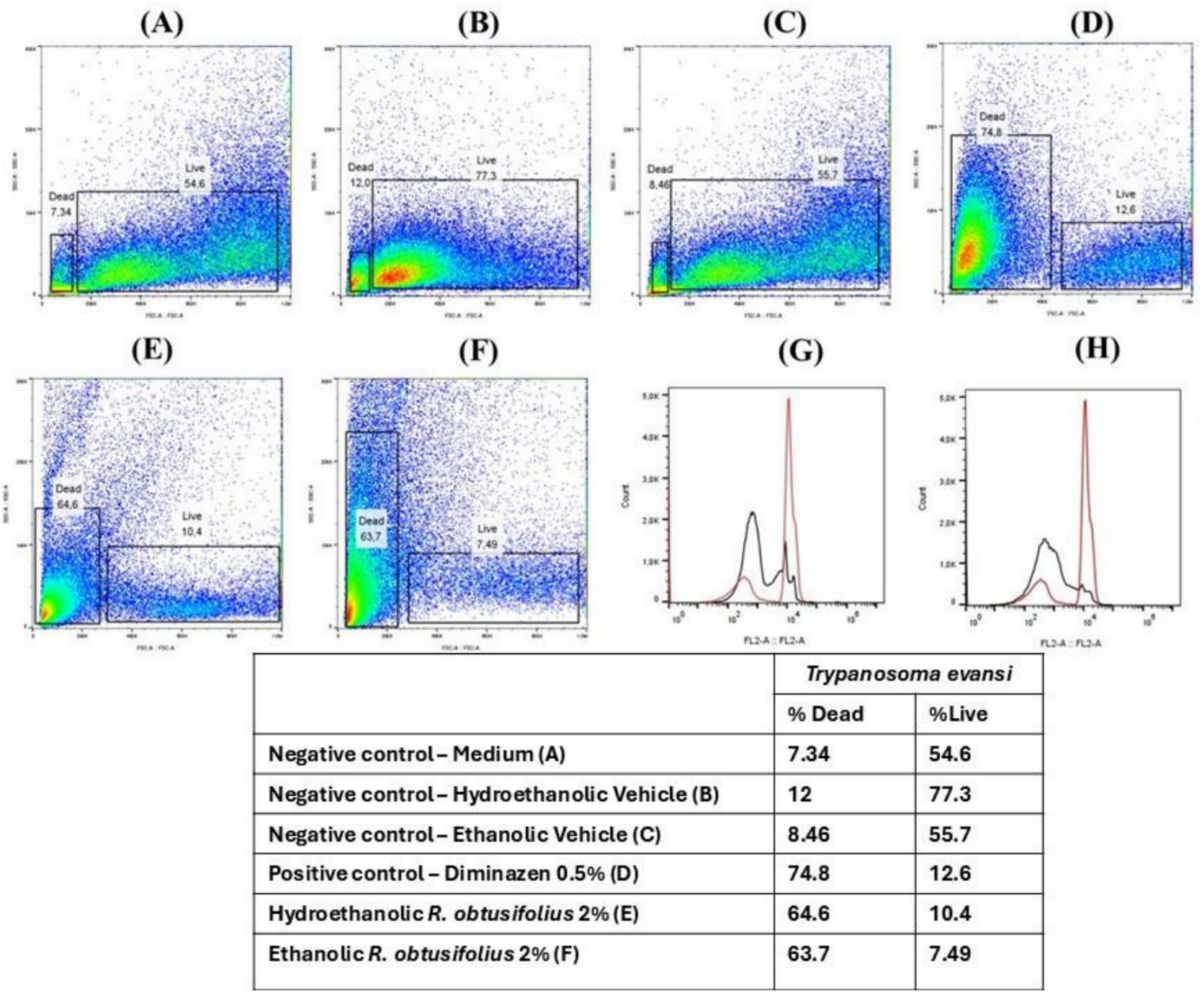
Plot SSC‐A/FSC‐A. (A) negative control: *T. evansi* without treatment. (B and C) *T. evansi* treated with hydroethanolic and ethanolic vehicles, respectively. (D) positive control: *T. evansi* treated with 0.5% aceturate diminazene; (E and F): *T. evansi* treated with 2% hydroethanolic and ethanolic extracts of leaves, respectively; (G and H): Overlapping of *T. evansi* treated with diminazene aceturate (DA) and extracts of leaves of *R. obtusifolius* . The parameters for analyses were Count/FL2‐A, where (G) DA and hydroethanolic extract of leaves of *R. obtusifolius* and (H) DA and ethanolic extract of leaves of *R. obtusifolius* . The table below the graphs summarizes the percentage of dead and live parasites.

The flow cytometry analyses of *T. evansi* treated with hydroethanolic and ethanolic extracts, as well as the positive control (0.5% diminazene aceturate) are shown in Figure 4. The negative controls with medium (Figure 4A), hydroethanolic (Figure 4B), ethanolic (Figure 4C) vehicles in flow cytometry analyses show a similar percentage of live (54.6%, 77.3% and 55.7%, respectively) and dead *T. evansi* population (7.34%, 12% and 8.46%, respectively). Diminazene aceturate (Figure 4D) showed the anti‐ *T. evansi* activity with 74.8% dead parasites. The 2% hydroethanolic (Figure 4F) and ethanolic (Figure 4G) leaves extracts demonstrated significant necrotic anti‐*T evansi* in vitro activity (64.6% and 63.7%, respectively), compared to the medium (7.4%). Results were also similar to those of the positive control (diminazene aceturate, 74.8%). The analysis comparing the mean fluorescence positive control (red line) and mean fluorescence of hydroethanolic (Figure 4G, black line) and ethanolic extracts of leaf (Figure 4H, black line), indicated an overlap between dead parasites. The percentage data are summarized on the table below the graphs, in the same Figure 4.

### 3.4 In Vitro Cytotoxicity Assay

**FIGURE 5:**
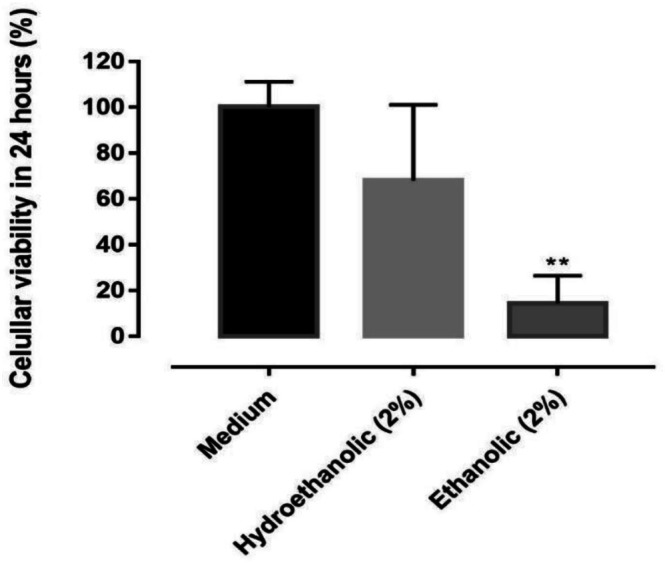
Cytotoxicity in Vero cells exposed to hydroethanolic and ethanolic extracts of leaf of *Rumex obtusifolius* , at concentrations of 2% for 24 h in MTT assay. Results expressed as % of cell viability. Differences between groups were assessed using the 1‐way ANOVA test and Bonferroni test (*), for values of *p* < 0.05.

Cell viability results by the MTT assay are shown in Figure 5. The ethanolic leaf extract (2%) reduced cell viability to 14.3% after 24 h, statistically significant if compared to the culture medium, which maintains the viability near 100%. The hydroethanolic extract at 2%, in turn, showed lower cytotoxicity, whose cell viability was at 65%–70% after 24 h, being statistically similar to the control group (*p* > 0.05).

### 3.5 Antibacterial Activity

**FIGURE 6:**
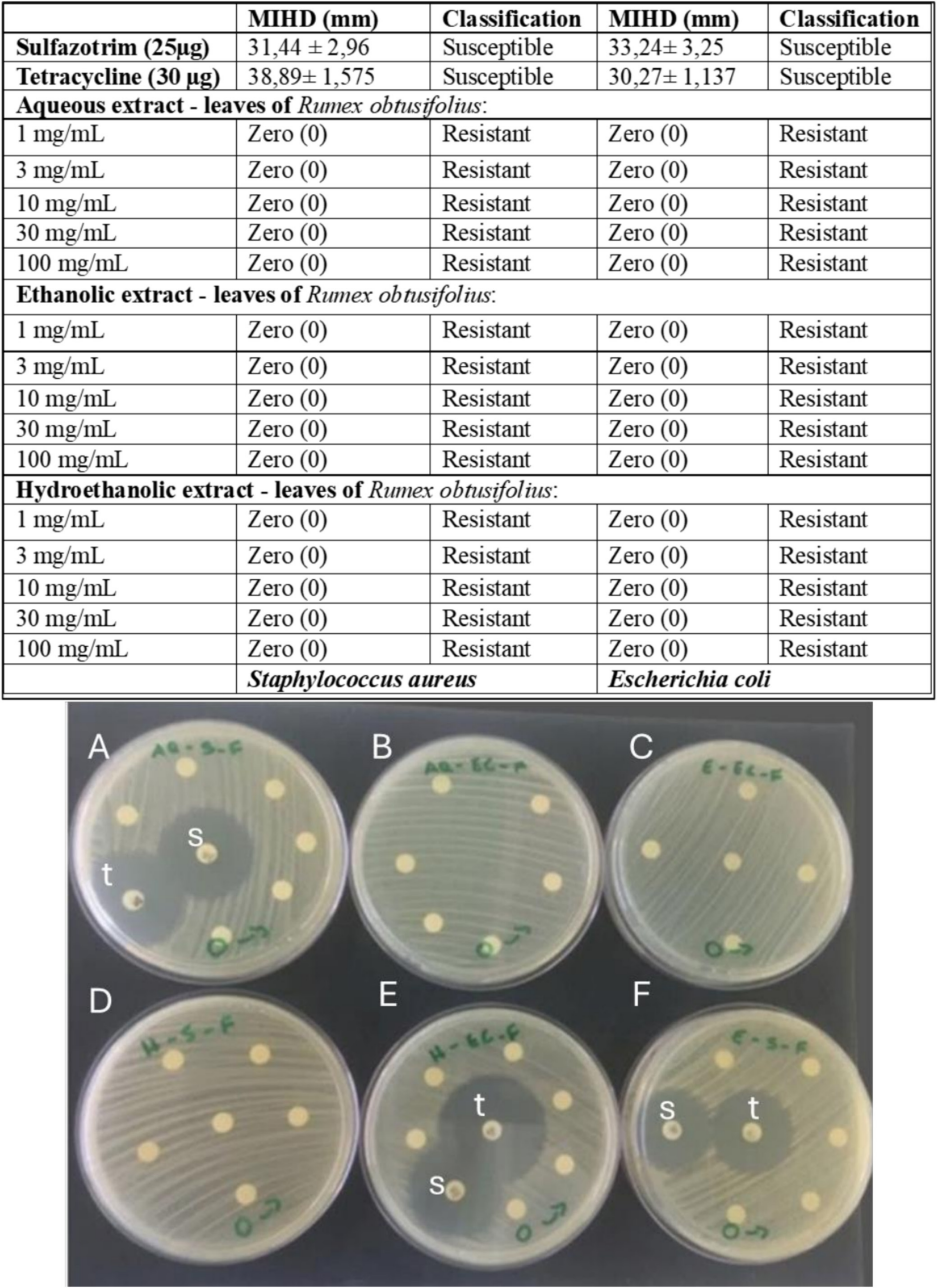
Antibacterial activity of aqueous (A and B), hydroethanolic (D and E) and ethanolic (C and F) extracts of leaf of *Rumex obtusifolius* , in different concentrations, against *E. coli* (B, C and E) and *S. aureus* (A, D and F) cultures. The table shows the median inhibitory halo diameter, in milimetres, and the classification of the agent, according to EUCAST and BrCAST [26, 27]. The image shows some examples from the experiment. The major circles are from the standard antibiotics sulfazotrim (s) and tetracycline (t).

The results obtained from the microbiological analyses against bacteria are shown in Figure 6, where it is possible to visualize the formation of an inhibition halo only on the positive control samples; therefore, no antimicrobial effect was observed for all concentrations of the studied extract.

## 4 Discussion

The results of the present study report, for the first time, the antitrypanosomal activity exhibited by hydroethanolic and ethanolic leaf extracts of *R. obtusifolius* against *T. evansi*, by inducing necrosis in such parasites. This action does not seem to be dependent on the antioxidant capacity of the plant. Moreover, this species could not show antibacterial activity against *Staphylococcus aureus* nor *Escherichia coli* .

*R. obtusifolius* belongs to the Polygonaceae family, with annual, biennial and perennial herb species cultivated in almost every acidic soil [28, 29]. Some studies have shown significant antioxidant activity in the roots and leaves of *R. obtusifolius* , in addition to bioactive compounds [16, 19, 29, 30]. Nonetheless, only a few studies have reported its chemical and pharmacological properties.

In this study, phytochemical and antioxidant analyses demonstrated that root extracts of *R. obtusifolius* exhibited significantly higher antioxidant activity than leaf extracts. The hydroethanolic root extract was the most active, showing the highest values for ABTS, FRAP and TPC, being significantly higher than all other extracts. Although root extracts presented superior antioxidant capacity overall, root samples did not exhibit anti‐ *T. evansi* activity.

Leaf extracts showed lower antioxidant activity than root extracts. Among leaf samples, the ethanolic extract displayed the highest FRAP value, followed by the hydroethanolic extract. The ethanolic leaf extract also exhibited significantly higher TPC and TFC compared with the hydroethanolic extract.

Regarding the total antioxidant activity by the DPPH, ABTS and FRAP methods, as shown in Table 1, in general, different extracting solvents had different rates of antioxidant activity. Overall, extracts with higher phenolic contents consistently showed higher antioxidant capacity, as evidenced by the positive association between TPC and FRAP/ABTS values, indicating that phenolic compounds may be major contributors to the antioxidant activity of *R. obtusifolius* . In agreement with these findings, it was reported that the *R. obtusifolius* leaf possesses high phenolic (129 ± 9 mg GAE. g^−1^ DW) and flavonoid contents (92 ± 4 mg RE. g^−1^ DW), placing this species among those with the richest phenolic profiles within the genus, despite differences in the basis of expression [31].

It is difficult to compare these data with the present ones, because ours are based on total weight and not dry weight (DW). Our values were about 20 times lower than the cited article. Considering the above, we hypothesize that antioxidant activity is not necessarily the primary mechanism underlying the antiparasitic effects of this plant species, as the *R. obtusifolius* root extract exhibited in vitro antioxidant activity but did not show trypanocidal activity.

Regarding antimicrobial activity, we can no longer say whether or not there is a relationship with antioxidant potential. Alterations in metabolic pathways mediated by the action of *R. obtusifolius* bioactive compounds, including the downregulation of carbohydrate transport and/or utilization as well as energy metabolism of some bacteria, were associated with antibacterial activity [32].

Literature indicates that *R. obtusifolius* contains a diverse range of bioactive secondary metabolites, including proanthocyanidins, tannins, quercetin, kaempferol, gallic acid and hydroxybenzoic acid derivative [31, 33]. These classes of compounds have been widely associated with antiparasitic properties and may therefore contribute to the antitrypanosomal activity observed for *R. obtusifolius* [34, 35, 36]. Our study showed that the hydroethanolic and ethanolic leaf extracts of *R. obtusifolius* exhibited significant in vitro antitrypanosomal activity against *T. evansi* , as demonstrated by parasite counting and flow cytometry analysis. At a concentration of 2%, both extracts achieved complete parasite elimination within 3 h, showing an efficacy comparable to the positive control, whereas 1% and 0.5% resulted in complete elimination at 6 and 9 h, respectively.

Hydroethanolic and ethanolic extracts from the leaf of *R. obtusifolius* were capable of inducing necrotic death of trypanosomes, as observed in the flow cytometry analysis results. This assay is also an important point of the results, since a few reports show this correlation. It is extremely important to explore other mechanisms of cell death in the development of new drugs and bioactive compounds with trypanocidal activity, as well as the pharmacokinetics and pharmacodynamics of plant species extracts obtained in the study. Actions on apoptosis, inflammatory mediators and oxidative stress of the parasite may be important targets to be investigated. The importance of other cell death mechanisms, such as apoptosis, inflammatory mediators and oxidative stress, that were not investigated in the present study cannot be ruled out.

According to the literature, the genus *Rumex* is already known for its antiparasitic properties. The ethanolic extract from the root of *Rumex crispus* was able to inhibit 50% of the viability of *Trypanosoma brucei* [37]. Tewabe et al. tested the in vitro and in vivo trypanocidal action of rein and alloin, important anthraquinones of Rumex genus, in mice infected with *Trypanosoma congolense* [38]. They presumed that these two bi vivo experiments showed that aloin doses of 200 and 400 mg/kg were sufficient to significantly reduce parasitemia in animals.

Woku et al. [39] evaluated a methanolic extract of *Rumex abyssinicus* against *T. brucei* , with the best results in vitro under a concentration of 333 ug/mL. The cytotoxicity in mammalian cells was not performed. The chloroform and ethyl acetate extracts from *R. crispus* have also exhibited significant antiplasmodial activity against *P. falciparum*, irrespective of chloroquine sensitivity or resistance. Nepodin A was isolated, identified as the active compound and also demonstrated potent antiplasmodial effects, showing lower cytotoxicity in Vero cells compared to chloroquine. Furthermore, this compound was effective in in vivo assays [40].

Furthermore, this plant genus and its anthraquinones have demonstrated antileishmanial activity from rhizome extracts [41] as well as antimalarial properties [40]. Other studies also demonstrated the activity of *R. obtusifolius* extracts against *Acanthamoeba* spp. [12].

Although antimicrobial activity against *E. coli* and *S. aureus* was not detected in the present study, this biological property has been consistently described in the literature, thereby supporting the antimicrobial potential of *R. obtusifolius* and Rumex genus [18, 42]. Antibacterial activity is strongly associated with the extraction of specific metabolites; depending on the solvent used, the extraction of these compounds may have been insufficient [43]. The solubility of these compounds in a specific solvent is a peculiar characteristic of phytochemicals [44].

An ethanolic leaf extract of *R. obtusifolius* exhibited antibacterial activity against *S. pyogenes* at concentrations from 0.05 to 0.40 g/mL, without cytotoxicity. In contrast, *S. aureus* and *E. coli* were resistant to the extract [45]. These findings are in agreement with our results. Another study has used a concentration of 200 μg/mL of the ethanolic extract from the roots of *R. obtusifolius* and showed antibacterial activity against *S. aureus* [46].

There is no information in the literature on the cytotoxicity of *R. obtusifolius* in mammalian cells. In our study, treatment with the 2% ethanolic extract from leaves of *R. obtusifolius* resulted in cytotoxic effects in Vero cells, with cell viability decreasing to 14.3% after 24 h relative to the culture medium control. This result needs to be considered in future in vivo approaches, and testing more extract concentrations and solvents.

The use of the highest dose in toxicological tests is encouraged in many official guidelines, such as those of the OECD [47]. However, the importance of concentration‐response curves is understood, and this is a bias in our results, especially since compounds can exhibit different concentration‐response curve patterns [48]. We believe that such results are valuable for understanding, in part, the toxicity of the *R. obtusifolius* extract.

The cytotoxicity of other *Rumex* species has already been reported. For example, the ethyl acetate root extract of *R. crispus* exhibited higher cytotoxicity against mouse leukaemic monocyte–macrophage cells, reducing cell viability to 60.20%, 2.62% and 17.33% at concentrations of 100, 200 and 400 μg/mL, respectively, whereas the ethanolic extract showed no cytotoxic effects [49]. The bioactive compounds from the ethyl acetate extract of *R. japonicus* Houtt. roots were isolated [50], and compounds such as emodin, aloe‐emodin, physcion and resveratrol exhibited cytotoxic activity against HepG2 cells. The cytotoxicity of aqueous, methanolic and chloroformic extracts from *R. crispus* (entire plant) using MTT in cell lineages of human carcinoma obtained an IC_50_ from 130 μg/mL [51]. The genus Rumex is also studied in cancer models precisely because of its cytotoxic potential, as we observed in our results as well. A systematic review confirms this for the species *R. abyssinicus*, commenting that emodin, physcion, chrysophanol and methyl gallate are the compounds that have been highly investigated for their pharmacological effect [52]. This is a good reason to explore the potential of the species, taking care of the possible adverse effects it could take.

The antitrypanosomal activity observed highlights the veterinary relevance of these findings, given the impact of animal trypanosomiasis on livestock health [53]. The efficacy and optimal feeding level of *Rumex nervosus* leaves on anticoccidial indicators of broiler chickens infected or not with *Eimeria tenella* were tested, with expressive results [54], being a good example of the practical use of this plant genus against diseases caused by protozoa in veterinary medicine.

Although our in vitro results suggest therapeutic potential, their applicability is limited by the absence of in vivo validation, pharmacokinetic data and safety assessments. Ntemafack et al. [52] have also pointed out this limitation in a majority of the studies involving another *Rumex* species. Consequently, further studies are required to confirm the feasibility of these extracts as veterinary treatments. Future research should advance through in vivo validation and subsequent clinical evaluation, ensuring a rigorous assessment of efficacy and safety.

## 5 Conclusion

The present study provides the first evidence of the in vitro antitrypanosomal activity of *R. obtusifolius* against *T. evansi* . The hydroethanolic and particularly the ethanolic leaf extract demonstrated significant efficacy at a concentration of 2%, with parasite elimination comparable to the reference drug (diminazene aceturate). Flow cytometry analyses confirmed that parasite death occurred predominantly via necrosis, offering preliminary insight into the possible mechanism of action, although further investigations are required for its full elucidation.

## Funding

The Article Processing Charge for the publication of this research was funded by the Coordenação de Aperfeiçoamento de Pessoal de Nível Superior—Brasil (CAPES).

## Conflicts of Interest

The authors declare no conflicts of interest.

## Data Availability

The data that support the findings of this study are available from the corresponding author upon reasonable request.

## References

[1] J. Kim , A. Álvarez‐Rodríguez , Z. Li , M. Radwanska , and S. Magez , “Recent Progress in the Detection of Surra, a Neglected Disease Caused by *Trypanosoma evansi* With a One Health Impact in Large Parts of the Tropic and Sub‐Tropic World,” Microorganisms 12, no. 1 (2024): 44, 10.3390/microorganisms12010044. PMC10819111 38257871

[2] G. Greif , P. Faral‐Tello , C. Scardoelli Vianna , A. Hernandez , Y. Basmadjian , and C. Robello , “The First Case Report of Trypanosomiasis Caused by Trypanosoma Evansi in Uruguay,” Veterinary Parasitology: Regional Studies and Reports 11 (2018): 19–21, 10.1016/j.vprsr.2017.11.002. 31014612

[3] N. Z. I. Mohd Rajdi , M. A. Mohamad , L. P. Tan , et al., “First Case Report on the Occurrence of *Trypanosoma evansi* in a Siam B Mare in Kelantan, Malaysia,” Veterinary Medicine and Science 7, no. 2 (2021): 303–309, 10.1002/vms3.379. 33161648 PMC8025636

[4] O. D. Salvioni Recalde , S. Fraenkel , M. José Tintel , et al., “First Report of the Presence of *Trypanosoma evansi* in Dogs From Paraguay Applying Molecular Techniques,” Brazilian Journal of Veterinary Medicine 43, no. 1 (2021): e001920, 10.29374/2527-2179.bjvm001920.

[5] P. P. Joshi , V. R. Shegokar , R. M. Powar , et al., “Human Trypanosomiasis Caused by Trypanosoma Evansi in India: The First Case Report,” American Journal of Tropical Medicine and Hygiene 73, no. 3 (2005): 491–495. 16172469

[6] R. M. Powar , V. R. Shegokar , P. P. Joshi , et al., “A Rare Case of Human Trypanosomiasis Caused by Trypanosoma Evansi,” Indian Journal of Medical Microbiology 24, no. 1 (2006): 72–74, 10.4103/0255-0857.19904. 16505565

[7] N. Van Vinh Chau , L. Buu Chau , M. Desquesnes , et al., “A Clinical and Epidemiological Investigation of the First Reported Human Infection With the Zoonotic Parasite *Trypanosoma evansi* in Southeast Asia,” Clinical Infectious Diseases: An Official Publication of the Infectious Diseases Society of America 62, no. 8 (2016): 1002–1008, 10.1093/cid/ciw052. 26908809 PMC4803109

[8] L. L. Kadosaki , S. F. de Sousa , and J. C. M. Borges , “Análise do Uso e da Resistência Bacteriana aos Antimicrobianos em Nível Hospitalar,” Revista Brasileira de Farmácia 93, no. 2 (2012): 129–133.

[9] A. Upadhyay , I. Upadhyaya , A. Kollanoor‐Johny , and K. Venkitanarayanan , “Combating Pathogenic Microorganisms Using Plant‐Derived Antimicrobials: A Minireview of the Mechanistic Basis,” BioMed Research International 2014 (2014): 761741, 10.1155/2014/761741. 25298964 PMC4178913

[10] D. Sun , W. Gao , H. Hu , and S. Zhou , “Why 90% of Clinical Drug Development Fails and How to Improve It?,” Acta Pharmaceutica Sinica B 12, no. 7 (2022): 3049–3062, 10.1016/j.apsb.2022.02.002. 35865092 PMC9293739

[11] D. Berillo , M. Kozhahmetova , and L. Lebedeva , “Overview of the Biological Activity of Anthraquinons and Flavanoids of the Plant *Rumex* Species,” Molecules (Basel, Switzerland) 27, no. 4 (2022): 1204, 10.3390/molecules27041204. 35208994 PMC8880800

[12] T. Nayeri , F. Bineshian , F. Khoshzaban , A. D. Asl , and F. Ghaffarifar , “Evaluation of the Effects of Rumex Obtusifolius Seed and Leaf Extracts Against Acanthamoeba: An In Vitro Study,” Infectious Disorders Drug Targets 21, no. 2 (2021): 211–219, 10.2174/1871526520666200422111044. 32321413

[13] O. Orbán‐Gyapai , E. Liktor‐Busa , N. Kúsz , et al., “Antibacterial Screening of Rumex Species Native to the Carpathian Basin and Bioactivity‐Guided Isolation of Compounds From Rumex Aquaticus,” Fitoterapia 118 (2017): 101–106, 10.1016/j.fitote.2017.03.009. 28300698

[14] M. M. Quradha , A. M. F. Qahtan , K. B. Kushkhov , and R. A. Mukozheva , “Antioxidant Activity of Silver Nanoparticles Synthesized From Crud Methanolic Extract of *Rumex nervosus* ,” Adyghe International Scientific Journal 24, no. 1 (2024): 49–57, 10.47928/1726-9946-2024-24-1-49-57.

[15] F. Bineshian , N. Bakhshandeh , M. Freidounian , and H. Nazari , “Anti‐Candida and Antioxidant Activities of Hydroalcohlic Extract of Rumex Obtusifolius Leaves,” Pakistan Journal of Pharmaceutical Sciences 32, no. 3 (2019): 919–926. 31278700

[16] P. Feduraev , G. Chupakhina , P. Maslennikov , N. Tacenko , and L. Skrypnik , “Variation in Phenolic Compounds Content and Antioxidant Activity of Different Plant Organs From *Rumex crispus* L. and *Rumex obtusifolius* L. at Different Growth Stages,” Antioxidants 8, no. 7 (2019): 237, 10.3390/antiox8070237. 31340505 PMC6680865

[17] M. Bektašević , M. Oraščanin , and E. Šertović , “Biological Activity and Food Potential of Plants *Rumex crispus* L. and *Rumex obtusifolius* L.: A Review,” Technologica Acta—Scientific/Professional Journal of Chemistry and Technology 15, no. 1 (2022): 61–67.

[18] L. Moncayo‐Molina , C. M. Moncayo‐Rivera , I. Spengler , J. A. Pino , and J. O. Rojas‐Molina , “Chemical Composition, Antioxidant and Antimicrobial Activities of *Rumex obtusifolius* L. Essential Oil From the Highlands of Ecuador,” Journal of Essential Oil Bearing Plants 27, no. 4 (2024): 949–958, 10.1080/0972060X.2024.2383201.

[19] W. G. Sganzerla , R. Schmit , M. D. Melo , et al., “Rumex Obtusifolius Is a Wild Food Plant With Great Nutritional Value, High Content of Bioactive Compounds and Antioxidant Activity,” Emirates Journal of Food and Agriculture 31, no. 4 (2019): 315–320, 10.9755/EJFA.2019.V31.I4.1946.

[20] P. Tveden‐Nyborg , B. Yang , U. Simonsen , and J. Lykkesfeldt , “BCPT Perspectives on Studies Involving Natural Products, Traditional Chinese Medicine and Systems Pharmacology,” Basic & Clinical Pharmacology & Toxicology 135, no. 6 (2024): 782–785, 10.1111/BCPT.14109. 39617689

[21] AOAC International , Official Methods of Analysis of AOAC International, 18th ed. (Association of Official Analytical Chemists, 2006).

[22] J. Zhishen , T. Mengcheng , and W. Jianming , “The Determination of Flavonoid Contents in Mulberry and Their Scavenging Effects on Superoxide Radicals,” Food Chemistry 64, no. 4 (1999): 555–559, 10.1016/s0308-8146(98)00102-2.

[23] K. K. Misra , S. Roy , and A. Choudhury , “Biology of Trypanosoma (Trypanozoon) Evansi in Experimental Heterologous Mammalian Hosts,” Journal of Parasitic Diseases: Official Organ of the Indian Society for Parasitology 40, no. 3 (2016): 1047–1061, 10.1007/s12639-014-0633-1. 27605836 PMC4996246

[24] P. Tveden‐Nyborg , T. K. Bergmann , N. Jessen , U. Simonsen , and J. Lykkesfeldt , “BCPT 2026 Policy for Experimental and Clinical Studies,” Basic & Clinical Pharmacology & Toxicology 137, no. 6 (2025): e70159, 10.1111/bcpt.70159. 41339103

[25] C. B. Colpo , S. G. Monteiro , D. R. Stainki , E. T. B. Colpo , and G. B. Henriques , “Infecção Natural por Trypanosoma Evansi em Cães,” Ciência Rural 35, no. 3 (2005): 717–719, 10.1590/s0103-84782005000300038.

[26] EUCAST . (n.d.) EUCAST , https://www.eucast.org.

[27] BrCAST – Brazilian Committee on Antimicrobial Susceptibility Testing . n.d. BrCAST , https://brcast.org.br.

[28] F. O. Jimoh , A. A. Adedapo , and A. J. Afolayan , “Assessing the Polyphenolic, Nutritive and Biological Activities of Acetone, Methanol and Aqueous Extracts of *Rumex sagittatus* Thunb,” African Journal of Pharmacy and Pharmacology 4, no. 9 (2010): 629–635, http://www.academicjournals.org/ajpp.

[29] D. Harshaw , L. Nahar , B. Vadla , G. Saif‐E‐Naser , and S. Sarker , “Bioactivity of *Rumex obtusifolius* (Polygonaceae),” Archives of Biological Sciences 62, no. 2 (2010): 387–392, 10.2298/abs1002387h.

[30] M. C. Purohit , N. Singh , G. Kumar , R. Rawat , and M. Singh , “Investigation of In Vitro Antioxidant Potential of Methanolic Extract of Bark of *Prunus cornuta* and Root of *Rumex obtusifolius* ,” Plant Archives 21, no. 1 (2021): 73–77, 10.51470/plantarchives.2021.v21.no1.010.

[31] P. Feduraev , L. Skrypnik , S. Nebreeva , et al., “Variability of Phenolic Compound Accumulation and Antioxidant Activity in Wild Plants of Some *Rumex* Species (Polygonaceae),” Antioxidants 11, no. 2 (2022): 311, 10.3390/antiox11020311. 35204194 PMC8868549

[32] Y. Liu , L. Yang , P. Liu , Y. Jin , S. Qin , and L. Chen , “Identification of Antibacterial Components in the Methanol‐Phase Extract From Edible Herbaceous Plant *Rumex madaio* Makino and Their Antibacterial Action Modes,” Molecules 27, no. 3 (2022): 660, 10.3390/molecules27030660. 35163925 PMC8839378

[33] M. Ginovyan , H. Javrushyan , S. Hovhannisyan , et al., “5‐Fluorouracil and Rumex Obtusifolius Extract Combination Trigger A549 Cancer Cell Apoptosis: Uncovering PI3K/Akt Inhibition by In Vitro and In Silico Approaches,” Scientific Reports 14, no. 1 (2024): 14676, 10.1038/s41598-024-65816-5. 38918540 PMC11199614

[34] S. P. Da Costa , R. A. Schuenck‐Rodrigues , V. D. S. Cardoso , S. S. Valverde , A. B. Vermelho , and E. Ricci‐Júnior , “Therapeutic Potential of Bioactive Compounds From *Brugmansia suaveolens* Bercht. & J. Presl,” Nutrients 15, no. 13 (2023): 2912, 10.3390/nu15132912. 37447241 PMC10343851

[35] P. B. Lalthanpuii , L. Lalrosangpuii , and K. Lalchhandama , “In Vitro Investigation of the Antiparasitic Effects of a Pentacyclic Triterpene From the Toothache Plant on Intestinal Worms of Poultry,” Open Veterinary Journal 15, no. 3 (2025 Mar): 1387–1396, 10.5455/OVJ.2025.v15.i3.30. 40276185 PMC12017702

[36] N. Llurba Montesino , M. Kaiser , R. Brun , and T. J. Schmidt , “Busca por Atividade Antiprotozoária em Preparações Medicinais à Base de Ervas; Novas Pistas Naturais Contra Doenças Tropicais Negligenciadas,” Molecules 20, no. 8 (2015): 14118–14138, 10.3390/molecules200814118. 26248069 PMC6332118

[37] O. A. Idris , O. A. Wintola , and A. J. Afolayan , “Evaluation of the Bioactivities of *Rumex crispus* L. Leaves and Root Extracts Using Toxicity, Antimicrobial, and Antiparasitic Assays,” Evidence‐Based Complementary and Alternative Medicine: eCAM 2019 (2019): 6825297, 10.1155/2019/6825297. 31827556 PMC6885263

[38] Y. Tewabe , D. Bisrat , G. Terefe , and K. Asres , “Antitrypanosomal Activity of Aloin and Its Derivatives Against *Trypanosoma congolense* Field Isolate,” BMC Veterinary Research 10, no. 1 (2014): 1–7, 10.1186/1746-6148-10-61/TABLES/4. 24612613 PMC3984735

[39] N. Worku , A. Mossie , A. Stich , et al., “Evaluation of the In Vitro Efficacy of Artemisia Annua, Rumex Abyssinicus, and Catha Edulis Forsk Extracts in Cancer and Trypanosoma Brucei Cells,” ISRN Biochemistry 2013 (2013): 910308, 10.1155/2013/910308. 25937964 PMC4392988

[40] K. H. Lee and K. H. Rhee , “Antimalarial Activity of Nepodin Isolated From Rumex Crispus,” Archives of Pharmacal Research 36, no. 4 (2013): 430–435, 10.1007/s12272-013-0055-0. 23440579

[41] S. T. Guetchueng , P. T. Djouonzo , Y. Lame , et al., “Antileishmanial Anthraquinones From the Rhyzomes of *Rumex abyssinicus* Jacq (Polygonaceae),” Natural Product Research 37, no. 17 (2023): 2935–2939, 10.1080/14786419.2022.2137797. 36282890

[42] M. Wegiera , U. Kosikowska , A. Malm , and H. Smolarz , “Antimicrobial Activity of the Extracts From Fruits of *Rumex* L. Species,” Open Life Sciences 6, no. 6 (2011): 1036–1043, 10.2478/s11535-011-0066-0.

[43] J.‐E. Lee , J. T. M. Jayakody , J.‐I. Kim , et al., “The Influence of Solvent Choice on the Extraction of Bioactive Compounds From Asteraceae: A Comparative Review,” Food 13, no. 19 (2024): 3151, 10.3390/foods13193151. PMC11475975 39410186

[44] F. J. M. Novaes , D. C. de Faria , F. Z. Ferraz , and F. R. de Aquino Neto , “Hansen Solubility Parameters Applied to the Extraction of Phytochemicals,” Plants 12, no. 16 (2023): 3008, 10.3390/plants12163008. 37631219 PMC10459436

[45] H. Koochak , S. M. Seyyednejad , and H. Motamedi , “Preliminary Study on the Antibacterial Activity of Some Medicinal Plants of Khuzestan (Iran),” Asian Pacific Journal of Tropical Medicine 3, no. 3 (2010): 180–184, 10.1016/S1995-7645(10)60004-1.

[46] R. Verma and S. Puri , “Antimicrobial, Antioxidant and GC‐MS Profiling of *Rumex obtusifolius* L. an Important Ethnomedicinal Plant of Himachal Pradesh in North Western Himalaya,” Medicinal Plants ‐ International Journal of Phytomedicines and Related Industries 8, no. 3 (2016): 249, 10.5958/0975-6892.2016.00030.7.

[47] OECD . 2025. Guidance on Grouping of Chemicals, OECD Series on Testing and Assessment OECD Publishing No. 418 3rd ed. 10.1787/b254a158-en.

[48] L. Yang , J. Wang , R. A. Cheke , and S. Tang , “A Universal Delayed Difference Model Fitting Dose‐Response Curves,” Dose‐Response: A Publication of International Hormesis Society 19, no. 4 (2021): 15593258211062785, 10.1177/15593258211062785. 34987337 PMC8689633

[49] T. Eom , E. Kim , and J. S. Kim , “In Vitro Antioxidant, Antiinflammation, and Anticancer Activities and Anthraquinone Content From *Rumex crispus* Root Extract and Fractions,” Antioxidants 9, no. 8 (2020): 726, 10.3390/antiox9080726. 32784977 PMC7464605

[50] Z. Wu , K. Ameer , and G. Jiang , “Isolation and Characterization of Anti‐Tumor Compounds From Ethyl Acetate Extract of Rumex Japonicus Houtt Roots and Their Cytotoxic Effects,” Food Science and Technology 42 (2022), 10.1590/fst.05421.

[51] B. Tüzün , “Evaluation of Cytotoxicity, Chemical Composition, Antioxidant Potential, Apoptosis Relationship, Molecular Docking, and MM‐GBSA Analysis of Rumex Crispus Leaf Extracts,” Journal of Molecular Structure 1323 (2025): 140791, 10.1016/j.molstruc.2024.140791.

[52] A. Ntemafack , M. Ayoub , Q. P. Hassan , and S. G. Gandhi , “A Systematic Review of Pharmacological Potential of Phytochemicals From Rumex Abyssinicus Jacq,” South African Journal of Botany 154 (2023): 11–25, 10.1016/j.sajb.2023.01.013.

[53] S. H. Pereira , F. P. Alves , and M. Ribeiro , “Animal Trypanosomiasis: Challenges and Prospects for New Vaccination Strategies,” Microorganisms 12, no. 12 (2024): 2575, 10.3390/microorganisms12122575. 39770779 PMC11678697

[54] A. H. Alqhtani , M. M. Qaid , S. I. Al‐Mufarrej , M. Al‐Garadi , and A. Pokoo‐Aikins , “Efficacy and Optimal Feeding Level of Rumex Nervosus Leaves on Blood Biochemistry, Carcass Characteristics, Productivity Indices, and Anticoccidial Indicators of Broiler Chickens Infected or Not Infected With Eimeria Tenella,” Brazilian Journal of Poultry Science (2023), 10.1590/1806-9061-2022-1786.

